# Probing three-dimensional collective cancer invasion with DIGME

**DOI:** 10.1186/s41236-017-0004-9

**Published:** 2017-11-01

**Authors:** Amani A. Alobaidi, Bo Sun

**Affiliations:** Department of Physics, Oregon State University, Weniger Hall, Corvallis, OR USA

**Keywords:** Tumor organoid, ECM, Collective migration

## Abstract

**Background:**

Multicellular pattern formation plays an important role in developmental biology, cancer metastasis and wound healing. While many physical factors have been shown to regulate these multicellular processes, the role of ECM micro-to-meso scale geometry has been poorly understood in 3D collective cancer invasion.

**Results:**

We have developed a mechanical-based strategy, Diskoid In Geometrically Micropatterned ECM (DIGME). DIGME allows easy engineering of the shape of 3D tissue organoid, the mesoscale ECM heterogeneity, and the fiber alignment of collagen-based ECM all at the same time. We have employed DIGME to study the 3D invasion of MDA-MB-231 diskoids in engineered collagen matrix. We find that the collective cancer invasion is closely regulated by the micro-to-meso scale geometry of the ECM.

**Conclusions:**

We conclude that DIGME provides a simple yet powerful tool to probe 3D dynamics of tissue organoids in physically patterned microenvironments.

**Electronic supplementary material:**

The online version of this article (doi:10.1186/s41236-017-0004-9) contains supplementary material, which is available to authorized users.

## Background

Invasion in three-dimensional (3D) extracellular matrix (ECM) is an important step in the lethal metastasis of tumors (Hunter et al. 2008). Although extensive studies have elucidated detailed mechanisms of single cell 3D motility (Friedl and Bröcker 2000; Grinnell and Petroll 2010; Petrie et al. 2012), and cell-ECM interactions (Bergert et al. 2012; Petrie and Yamada 2013; Doyle et al. 2015), 3D collective cancer invasion is still poorly understood (Christiansen and Rajasekaran 2006; Friedl and Gilmour 2009; Friedl et al. 2012; Carey et al. 2015). Most studies to date have focused on 2D collective cell migration. It has been shown that cell-cell adhesion (Maruthamuthu et al. 2011; Bi et al. 2015; Bazelliéres et al. 2015), exclusion volume (Angelini et al. 2011), contact inhibition (Liu et al. 2011; Zimmermann et al. 2016), cell-secreted chemical factors (Theveneau et al. 2013), and substrate-mediated mechanical forces (Ma et al. 2013) coordinate the multicellular motility and pattern formation of multiple cells in 2D. These results, however, have limited applicability in 3D tumor progression. The topological connectivity and porosity of 3D ECM allow cells to avoid touching one another while migrating, thus mechanical signaling via direct cell-cell contact is less important for collective motion in 3D than in 2D (Liu et al. 2013; Liang et al. 2016). Similarly, chemical signaling in 3D suffers from rapid dispersive dilution, thus the diffusion-mediated 3D intercellular correlations are much weaker compared to the case in 2D (Sun et al. 2012).

To probe the collective cell migration and morphogenesis, 2D cell patterning and substrate engineering has provided much insights. For instance, various types of wound healing assays have been developed to explore the invasion of cancer cell colonies into extracellular voids of pre-defined geometries (Poujade et al. 2007; Brugués et al. 2014; Ravasio et al. 2015). These assays typically use soft-lithography fabricated stamps when seeding the cells, and lift the stamps after the cells have adhered to the substrate. Alternatively, geometric patterned cell adhesives and cell repellents (Singhvi et al. 1994; Rape et al. 2011; Deforet et al. 2014), as well as microfluidics channels (Huang et al. 2011) have been employed to restrict cell migration. By engineering the confining geometry of the substrate, emergent multicellular dynamics, such as spontaneous rotation in circular geometry (Notbohm et al. 2016), and directed migration in ratchet geometry (Mahmud et al. 2009) have been observed.

To probe 3D collective cancer invasion, multicellular tumor spheroid models have been widely employed (Inch et al. 1970; McCredie et al. 1971; Hirschhaeuser et al. 2010). Tumor spheroids are aggregates of cancer cells that preserve native 3D cell-cell contact, mimicking the configuration of solid tumors (Weiswald et al. 2015). Multiple methods have been developed to grow 3D tumor spheroids, such as the hanging droplets (Timmins and Nielsen 2007), non-adhesive microplates (Gong et al. 2015), and bi-phase liquid systems (Han et al. 2015). However, these techniques can neither control the geometry of the cell aggregates, nor have the capability of engineering complex extracellular environment. Other methods, such as 3D tissue printing (Villar et al. 2013) and photo-sensitive hydrogel (Hahn et al. 2006; Kloxin et al. 2009; DeForest and Anseth 2011; Applegate et al. 2015) are capable of 3D cell-ECM patterning at the price of expensive equipment, non-native ECM composition, or sophisticated sample preparation (Yanagawa et al. 2016). As an alternative, we extend the mold-based technique developed by Nelson et al. (2006) into a low-cost, flexible strategy, Diskoid In Geometrically Micropatterned ECM (DIGME). DIGME is mechanical-based, and is compatible with a wide range of cells and ECM types. As we will demonstrate below, DIGME combines the powers of 3D tumor organoids and 3D ECM patterning, allowing us to independently control the shape of tumor organoids, microstructure and spatial heterogeneity of the ECM simultaneously.

## Methods

### Instrumentation

The basic setup of DIGME consists of a x-y-z translational stage to hold sample dish, and a rotational motor to mount the mold above the sample stage. We have used parts from Thorlabs Inc. and TA instruments to assemble prototypes of DIGME. When necessary, we have also placed the DIGME setup on an inverted microscope (Leica Microsystems) to help with alignment and positioning the mold. See Additional file 1: Section S1 for schematic design of the DIGME setup.

### Preparation of the collagen gel

High concentration collagen solution (10 mg/ml, Corning) is diluted and neutralized to desirable concentrations with cell growth medium (see cell culture), NaOH and 10X PBS, all purchased from Sigma-Aldrich. The neutralized, ice cold solution is first poured into the sample dish mounted on the DIGME setup where the mold is approximately 200 *μ*m above the glass bottom of the dish. After curing in the DIGME setup for 30–50 min (with the mold statically immersed or rotatin in the geling solution), we lift the mold out of the dish via the z-motor of the translational stage. The collagen solution will continue the gelation process for another 40 min. The molded gel is then immersed with fresh growth medium and stored at 4 °C for up to 2 days before adding cells.

### Cell culture and microscopy

GFP-labeled MDA-MB-231 cells (Cell Biolabs Inc.) are maintained according to the vendor’s protocol. After embedding the cells, DIGME devices are kept in tissue culture incubator except when taken out for imaging. For confocal imaging, we use a Leica SPE microscope. 10X oil immersion objective is used when confocal reflection imaging of collagen fiber is needed. Otherwise, 4X air objective is used to image the fluorescently labeled cells and fluorescent particles embedded in the collagen matrix. The z-stacks of confocal imaging are taken with 2 *μ*m z-steps. Confocal images are further processed in NIH ImageJ and MATLAB.

## Results

**Fig. 1:**
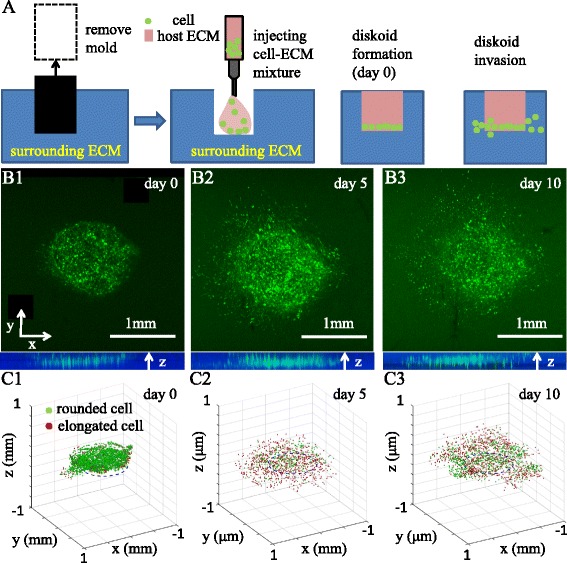
Preparation and collective migration of a circular DIGME. **A** Schematics showing the steps of forming a DIGME device. **B1**-**B3** Top views of a diskoid in 3D ECM. GFP-labeled MDA-MB-231 cells are cultured in DIGME device and confocal imaging are performed at day 1, 5 and 10. Bottom insets show the corresponding side views. **C1**-**C3** Manually identified 3D cell centers and morphological phenotypes corresponding to **B1**-**B3**. Green: rounded cells with aspect ratio less than 2. Red: elongated cells with aspect ratio greater than 2

To demonstrate the working principles and biocompatibility of DIGME, we first formed a cylindrical MDA-MB-231 tumor diskoid in 3D type-I collagen gel. Briefly, a stainless steel needle is used to mold the collagen gel (surrounding ECM) with a cylindrical well. The well is then filled with neutralized cell-collagen solution. After cells quickly sediment down to the well bottom (within 1 min), collagen solution continues to polymerize and eventually forms the host ECM that covers the cell aggregate – a diskoid – on the bottom of the well (Fig. 1 A). Within 24 h of incubation, cells start to invade the surrounding ECM. Figure 1 B1-B3 demonstrate the top views (x-y plane) and side views (x-z plane) of a DIGME sample at day 1, 5 and 10. Notice that although the surrounding ECM and the host ECM are polymerized at the same temperature (21 °C) and have the same concentration (1.5 mg/ml), the invasion in the radial direction is much more pronounced compared with the spreading in the z direction. The biased migration direction is presumably a collective phenomena due to the cell-cell interactions (Liu et al. 2013; Valencia et al. 2015).

DIGME allows continuous confocal imaging at the single cell level, therefore we can track the morphological profiles of the diskoid over time. Cells in the diskoid generally exhibit two distinct morphologies: elongated cells are typically fast moving and strongly contracting, while rounded cells migrate with short persistence and exert only weak traction forces (Giri et al. 2013; Kim et al. 2016). Empirically, we distinguish elongated and rounded cells based on the cell aspect ratio with a threshold value of 2. We have manually located the center of each cell and have classified each cell into elongated or rounded phenotypes as shown in Fig. 1 C1-C3.

**Fig. 2:**
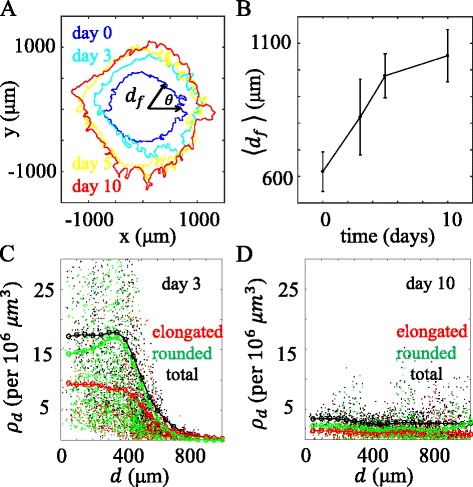
The invasion profile of a DIGME device. **a** The invasion fronts at day 0, 3, 5, and 10 of the same sample described in Fig. 1. **b** The means and standard deviations of the invasion distance obtained by averaging *d*_*f*_(*θ*) over the polar angle *θ* of **a**. **c**-**d** Scattered plots: the local cell density by counting only the elongated cells (red), or only the rounded cells (green), or all cells (black). Lines: Running average of the scattered data points with a Gaussian kernel. **c** shows the results at day 3, **d** shows the results at day 10

In order to quantify the diskoid invasion profile, we have located the invasion fronts by projecting the confocal images onto the x-y plane. The invasion front can be described as *d*_*f*_(*θ*,*t*), where *d*_*f*_ measures the radial distance from the center of the well, *θ* is the polar angle, and *t* is the time of diskoid invasion. Figure 2 a shows the invasion fronts at days 0, 3, 5, and 10. After averaging over the polar angle *θ*, Fig. 2 b shows the means and standard deviations of *d*_*f*_. The invasion front grows sublinearly with time, which is consistent with the observation on tumor spheroid (Valencia et al. 2015).

**Fig. 3:**
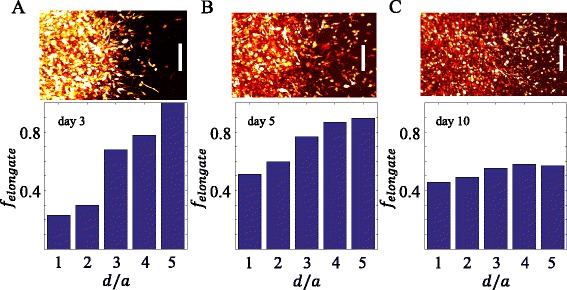
The spatial-temporal profiles of cell morphology. **a**-**c** Fraction of elongated cells *f*_*elongate*_ at varying distances from the center. Here the distance is normalized by the seeding radius *a*= 370 *μ*m of the same diskoid described in Fig. 1. Top insets: a section of the top view invading diskoid taken at days 3, 5 and 10. Scale bars of the insets: 200 *μ*m

In order to quantify the morphological evolution of the diskoid, we have calculated the elongated, rounded and full cell density using k-nearest neighbors of each cell. Briefly, for each cell *i* at location **r**_*i*_=[*x*_*i*_,*y*_*i*_,*z*_*i*_], we find the minimal sphere centered at **r**_*i*_ with radius *r*_*m*_ that encloses exactly k cells. The cell density at **r**_*i*_ is approximated to be $\rho _{d}(\mathbf {r}_{i}) = \frac {3k}{4\pi {r_{m}^{3}}}$. For simplicity, we have chosen *k*=10. Figure 2 c-d show the cell density at varying invasion depth *ρ*_*d*_(*d*), where $d=\sqrt {x^{2}+y^{2}}$. At day 3, only a small number of cells have migrated far from the seeding radius (original diskoid-ECM interface) at *a*= 370 *μ*m, and these cells are mostly elongated. Close to the center, cell density is approximately constant for *d*≤ 300 *μ*m. As invasion proceeds, the region of constant cell density expands. At the same time, cell density in this region decreases because the cell proliferation is slow compared to the migration-induced dilution. After invading the surrounding ECM for 10 days, both elongated and rounded cells are uniformly distributed for *d*≤ 1 mm, and the cell density has dropped by more than four folds. To further quantify the morphological distribution, we have normalized the invasion depth *d* with respect to the seeding radius *a* of the diskoid and have calculated the fraction of elongated cells *f*_*elongate*_ in different regions of *d*/*a*. As shown in Fig. 3 a, at day 3 *f*_*elongate*_ increases rapidly at greater radial distance, consistent with the fact that elongated cells are scout cells during collective invasion. The positive correlation between *f*_*elongate*_ and *d*/*a* gradually diminishes over time (Fig. 3 b). At day 10, a half-half mixture of elongated and rounded cells are found in all regions of the sample. To quantitatively account for these observations, we have developed a simple model based on persistent random walks. We assume the cells stochastically transform between elongated and rounded phenotypes at a rate of *τ*_*trans*_, and the two phenotypes have distinct migration persistent time *τ*_*el*_ and *τ*_*rd*_. As elaborated in the Additional file 1, the model agrees well with the results of Figs. 2 and 3.

**Fig. 4:**
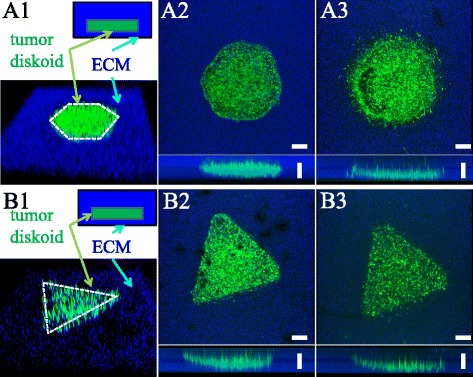
The hexagonal and triangular diskoid in DIGME devices. **A1** and (**B1**): 3D view of the diskoids. Blue: The surrounding ECM embedded with fluorescent particles. Green: GFP-labeled MDA-MB-231 cells. The seeding geometry of the diskoids are outlined in white. **A2**-**A3** Top and side views of the hexagonal diskoid at day 0 and day 5. **B2**-**B3**, Top and side views of the triangular diskoid at day 0 and day 5. Scale bars: 200 *μ*m

Solid tumors may develop various shapes in vivo, resulting in a diverse range of interfacial geometry between the cells and the ECM. DIGME allows us to easily control the geometry of diskoid. To demonstrate the capability, we employed laser-micromachining to fabricate stainless steel needles with hexagon and triangle cross-sections. Using these needles as the mold, we have generated hexagonal and triangular MDA-MB-231 diskoids in 3D collagen ECM (Fig. 4 A1 and B1). We find that the original shapes of the diskoid (Fig. 4 A2 and B2) largely determine the invasion pattern after 5 days of incubation (Fig. 4 A3 and B3). Previously it was reported that the interfacial geometry regulates the tumorigenicity by promoting cancer-stem cells (Lee et al. 2016). Using DIGME, we show that the geometric control can be realized in truly 3D setups.

**Fig. 5:**
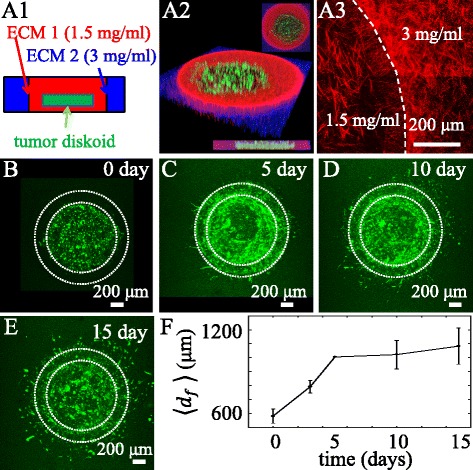
A two-layer DIGME device. **A1**: Schematics of the two-layer device. A circular MDA-MB-231 diskoid is confined by 1.5 mg/ml collagen matrix (ECM 1). ECM 1 is inside of 3 mg/ml collagen matrix (ECM 2). **A2**: 3D, top, and side views of the device. Green: MDA-MB-231 cells. Red: ECM 1 labeled with red fluorescent particles. Blue: ECM 2 labeled with far-red fluorescent particles. **A3**: confocal reflection image showing the collagen fibers at the interface of ECM 1 and ECM 2. **B**-**E** Top views of the invading diskoid at day 0, 5, 10 and 15. **F**: Invasion distance *d*_*f*_ as a function of time

**Fig. 6:**
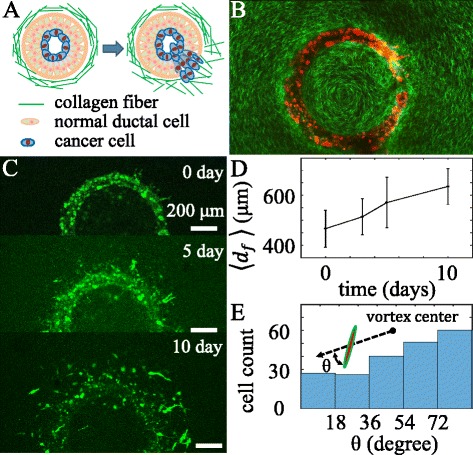
A ring diskoid in circularly aligned collagen matrix simulating a ductal carcinoma. **a** schematics showing the invasion of a typical ductal carcinoma. **b** A confocal slice showing the MDA-MB-231 ring diskoid (*red*) surrounded by circularly polarized collagen fibers (*green*). **c** Top views of the sample at day 0, 5, and 10. **d** Invasion distance *d*_*f*_ as a function of time. **e** Histogram of cell orientation *θ* at 10 days. *θ* is the angle between the cell long axis and the local radial direction measured from the seeding center of the diskoid *C*_*v*_

The extracellular space along the invasion path of a tumor is spatially heterogeneous (Shin et al. 2014). Employing DIGME, we can program the ECM heterogeneity and study its effect on the collective cancer invasion. As a proof of concept, we have formed a MDA-MB-231 diskoid confined within a two-layer ECM. This is done by sequentially applying two circular molds of different diameters, and filling the coaxial wells with different concentrations of collagen gels (Fig. 5 A1-A3). The inner layer, with collagen concentration 1.5 mg/ml is more porous compared to the outer layer, which has collagen at concentration of 3 mg/ml (Fig. 5 A3). We have observed the invasion of the diskoid for over 15 days, and the top views of the sample at days 0, 5, 10 and 15 are shown in Fig. 5 B-E. Within 5 days after initial seeding, the invasion front quickly reaches the interface of the inner and outer layer of the ECM (Fig. 5 C). At day 5, most front cells polarize tangentially at the interface, with a few leading cells polarizing radially and starting to invade the outer ECM layer. By quantifying the invasion front profiles, we find that that invasion speed is significantly reduced at day 5. Due to the change of cell orientation as well as the ECM microstructure, the invasion speed reduces rather abruptly at the interface between the two ECM layers. Ductal carcinoma is the most common type of breast cancer. At the early stage of ductal carcinoma, tumor cells are surrounded by a collagen matrix that are polarized long the tumor-stromal interface (Fig. 6 a) (Provenzano et al. 2006). The orientation of collagen fibers becomes disorganized even perpendicularly aligned during tumor progression, correlating with the clinical outcome of cancer patients (Conklin et al. 2011). Employing DIGME, we can control the orientation of collagen fibers in the surrounding matrix, mimicking different stages of ductal carcinoma. As an example, we mount a 150 *μ*m diameter needle approximately 300 *μ*m off the rotational axis of DIGME. While the 1.5 mg/ml collagen gel is forming, we continuously rotate the needle at 1 Hz for 5 min. The microscopic flow driven by the needle aligns the collagen fibers, and the fiber orientation is subsequently locked by the gelation process (Guo and Kaufman 2007). At the same time, the rotating needle carves a ring in the collagen gel, which we fill with MDA-MB-231 cells mixed in the host ECM (Fig. 6 b). The host ECM consists of 1.5 mg/ml collagen matrix that is randomly oriented. We have imaged the invasion process of the diskoid for 10 days, and find that the circularly polarized collagen fibers strongly impact the motility and morphology of MDA-MB-231 cells. The front invasion speed (Fig. 6 d) is noticeably slower compared with the diskoid in randomly oriented matrix (Fig. 2 b), and that a large fraction of cells are oriented tangentially along the collagen fibers (Fig. 6 e). These results are consistent with the contact guidance observed for single cells in both 2D and 3D cultures (Kim et al. 2012).

## Discussion and conclusion

We have described DIGME as a low-cost, easy-to-implement strategy to engineer the geometric microenvironment of tumor organoids. The shape-programmable organoid - diskoid, preserves the native cell-cell contacts in 3D ECM, and allow us to study the single cell dissemination and cohesive progression during collective cancer invasion.

For a thin circular diskoid in isotropic homogeneous ECM, we have shown that the collective invasion and morphological evolution of MDA-MB-231 cells (Figs. 1, 2 and 3) follow the similar patterns observed in the middle plane of tumor spheroids (Valencia et al. 2015). Tumor spheroids are often too dense to image through directly, and requires destructive pre-imaging preparations such as cryo-section. DIGME, on the other hand, provides an alternative model allowing continuous, long-term imaging at the single cell level.

We find that the invasion profile correlates with the seeding geometry, as shown in the hexagonal and triangular diskoids (Fig. 4). It has been proposed that physical forces generated by the cellular traction propagate over the ECM, and coordinate the 3D collective cancer invasion (Wang et al. 2014; Valencia et al. 2015; Liang et al. 2016). By controlling the shape of diskoids as well as the ECM microstructure, we can tune the stress distribution in the ECM. Therefore DIGME provides an ideal experimental system to understand the mechanical mechanisms coordinating the self-organized collective cancer invasion (Gjorevski et al. 2015).

We find that 3D collective cancer invasion is regulated by the spatial heterogeneity, as well as the microscopic anisotropy of the ECM. A progressing tumor encounters dramatically varying microenvironments, or microniches (Junttila and de Sauvage 2013). By employing DIGME, we can further extend the examples demonstrated in Figs. 5 and 6 to generate complex microniches in the ECM. For instance, epithelial cells and fibroblast cells can be embedded in different layers of ECM. Also the level of ECM fiber alignment can be controlled by varying the rotational protocol that drives the DIGME mold. Such protocols, including changing the rotational speed, or implementing bidirectional rotation, can be easily realized with programmable rotary motors.

In the above, we have used breast cancer cell line MDA-MB-231 cells to demonstrate the capabilities of DIGME. It is expected that DIGME methods equally apply to any cells compatible with 3D culture, such as fibroblast cells, endothelial cells, stem cells and neural cells. Similarly, type I collagen ECM can be replaced by other forms of ECM in DIGME, including tissue-derived ECM like matrigel, and synthetic ECM like peptide gel. With these extensions, DIGME is not only useful to probe the collective invasion of tumors, but also allows one to study 3D multicellular dynamics in wound healing, angiogenesis, development, and tissue remodeling.

We notice that the current form of DIGME has several limitations. First of all, DIGME only control the cross-sectional shape of the diskoid, rather than the full 3D geometry of the tumor organoid. Although variants of DIGME is possible, for instance by using a cone-shaped mold, fully 3D patterning may require incorporating other techniques such as directed self-assembly (Varner and Nelson 2014). Second, metal mold fabrication has a typical tolerance of tens of micrometers, or the size of a cell. In order to control the diskoid shape down to subcellular accuracy, one may incorporate other micro-fabrication techniques, such as polymer laser micromachining or deep reactive-ion etching (Liu et al. 2011). These alternative fabrication methods have micrometer *μ*m resolution, but may require surface treatment to ensure low-binding affinity to collagen. Third, the mechanical-based DIGME method requires one to two hours to prepare each sample. To improve the throughput, one may take advantage of the low-cost and simple operation of DIGME and implement automated parallel processing. Finally, DIGME patterns densely populated cells in 3D, which is different from the pathological case where the tumor is grown from a few transformed cells. Future in vivo studies will be required to validated the biological insights obtained from DIGME devices, such as the invasion dynamics and drug responses.

## Additional file

Additional file 1 Supplementary Information. (PDF 1700 kb)

## Abbreviations

DIGME: Diskoid in geometrically micropatterned ECM
ECM: Extracellular matrix
