# Supplementary material for: Probing three-dimensional collective cancer invasion with DIGME
Source: Cancer Converg. 2017 Nov 1;1(1):1. doi: 10.1186/s41236-017-0004-9 (PMC5876692; doi:10.1186/s41236-017-0004-9)
Supplement: Supplementary file 1 — Supplementary Information. (PDF 1700 kb) [file 41236_2017_4_MOESM1_ESM.pdf]

Supplementary Information

Amani A. Alobaidi[1] and Bo Sun*[1]

[1]*Department of Physics, Oregon State University, Corvallis OR, USA*

* sunb@onid.orst.edu

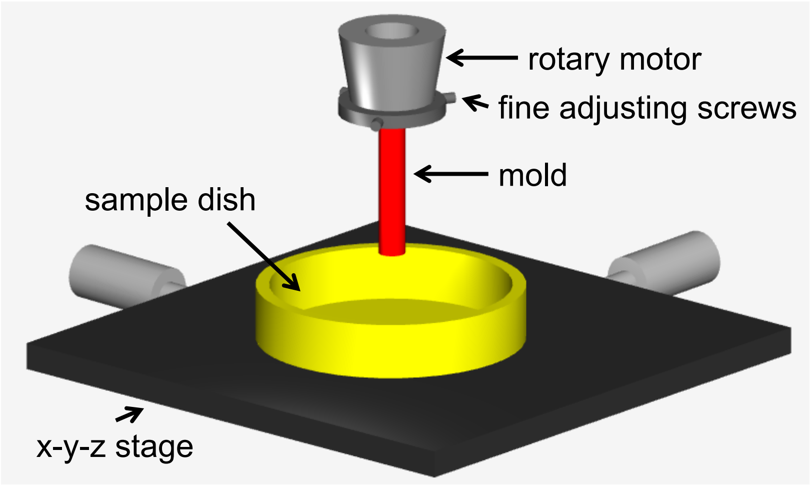

FIG. S1: Schematic design of experimental setup. The x-y axises of the stage position the sample dish with respect to the mold. The z-axis of the stage allows lifting up the mold from the partially-formed collagen gel. The fine adjusting screws position the mold with respect to the rotation axis of the rotary motor.

S1: ADDITIONAL INFORMATION OF EXPERIMENTAL SETUP

The schematic design of the mechanical setup is shown in Fig. S1.

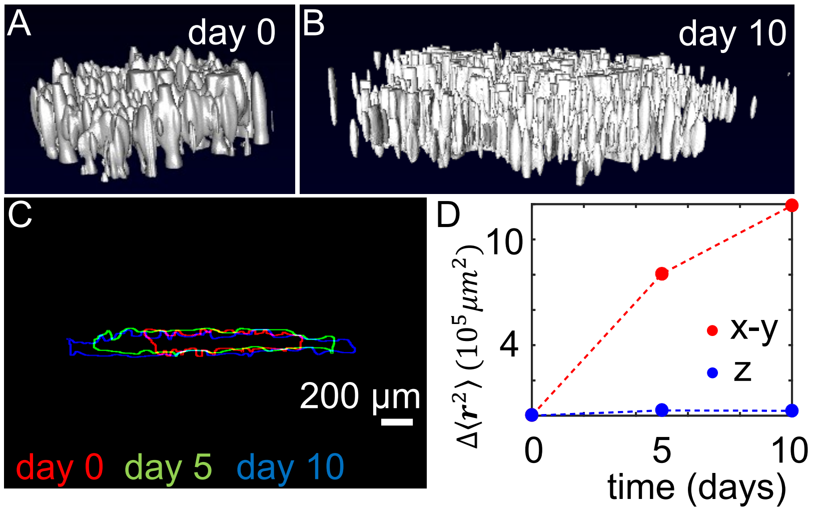

FIG. S2: 3D invasion profile of a typical circular diskoid. (A-B) 3D reconstruction of the cells on day 0 and day 10 from confocal image stacks. (C) Outlines of the invasion fronts at three time points from side view. (D) Changes of the mean square displacement $< \mathbf{r}^2 >$ in the $x - y$ plane (red) and in the z direction (blue). Here $\mathbf{r}$ is measured from the diskoid center.

S2: ADDITIONAL INFORMATION OF 3D DISKOID INVASION DYNAMICS

We find that for circular diskoids the invasion is mainly in the x-y plane. This is evident in the 3D reconstruction of the diskoid (Fig. S2 A-B), as well as the invasion fronts outlined from the side views (Fig. S2C). To further quantify the effects, we note that the approximate diffusion coefficients can be obtained from the mean square displacements of the cells. In particular, for N cells moving in d dimensions with effective diffusion coefficient D, we have

$$\Delta\langle \mathbf{r}^2 \rangle = \langle \mathbf{r}^2(t) \rangle - \langle \mathbf{r}^2(0) \rangle = 2dDt \tag{1}$$

From high resolution confocal images, we are able to obtain the 3D cell locations at various time points, and estimate the diffusion coefficients. In Fig. S2D, we plot $\Delta\langle \mathbf{r}^2 \rangle$ for the $x-y$ and z components separately, from which we find that the diffusion coefficient in the $x-y$ plane is 10 times higher than the diffusion coefficient in the z direction. Despite the fact that diffusive motion is only an approximation, the conclusion is clear that cancer invasion in the z direction is less pronounced compared with invasion in the x and y directions.

Combining high resolution confocal imaging and continuous 2D imaging, we can obtain more detailed cell dynamics. Fig. S3 shows an example of a subvolume in a circular diskoid. We find that the region corresponding to the original diskoid (R1) see a reduction in cell

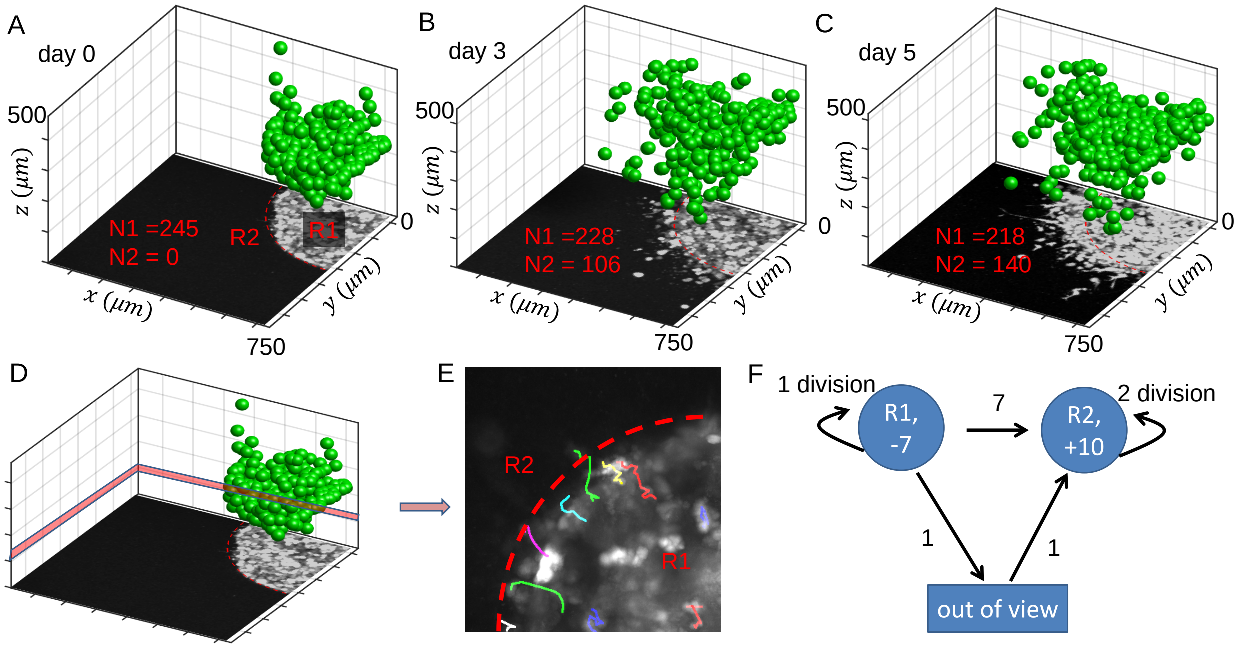

FIG. S3: Invasion dynamic of a subvolume in a circular diskoid. (A-C) The manually obtained cell locations based on high resolution confocal image stacks taken at day 0, 3, and 5 of a diskoid. The gray scale images are the maximum projections of the image stacks. We define two regions R1 and R2, corresponding to within and outside of the original diskoid. Region R1 and R2 are separated by red dashed line. N1 and N2 are cell counts within region R1 and R2. (D) In between day 0 and day 3, we have also taken 2-D time-lapse epifluorescent images, corresponding to a thin slice ($\approx$ 10 μm, set by the focal depth of 10x air objective) in the diskoid. (E) Trajectories of cells in the focal volume highlighted in (D) between day 0 and day 3. (F) Changes of cell number in R1 and R2 within the focal volume highlighted in (D) due to migration and cell division.

number, even in the presence of cell divisions. On the other hand, the region outside of the original diskoid gain significant number of cells. These observations are consistent with the density evolution as discussed in the main text. The confocal stacks and the continuous imaging video can be found at the online data repository associated with the manuscript.

To better visualize the packaging of the cells in the diskoid, we have prepared a circular diskoid under the same condition as the ones reported in the main text Fig. 1. We have fixed the cells and labeled the F-actin with fluorescent phalloidin (Fig. S4). The side view show that the cell diskoid contains approximately 4 cell layers after seeding.

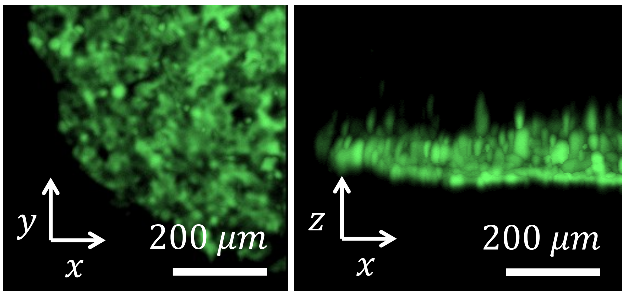

FIG. S4: The top view and side view of a MDA-MB-231 circular diskoid. The cells are fixed and F-actin has been labeled with immunofluorescence.

S3: STOCHASTIC SIMULATION OF DISKOID INVASION

We have developed a stochastic simulation model to quanlitatively explain the observations of the main text Fig. 2 and Fig. 3. The model assumes an independent, isotropic random walk by the cells in the real space, and a 1-D random walk in the parameter space so that phenotype switching (between rounded and elongated types) follows the first-passage kinetics. Rounded and elongated cells are assigned with different real space diffusion coefficients. With the assumption of isotropic migration, we only perform 2-D simulation for simplicity. The simulation algorithms is described in detail in below:

- The system is initialized so that $N = 5000$ cells are uniformly seeded in the unit circle with coordinates $\{x_i\}$, $\{y_i\}$. Each cell also has a phenotype coordinate $\{p_i = 0\}$. All cells are labeled as rounded cells.
- Advancing each time step, a rounded cell move by $dx_i = D_r G(0, 1)$, $dy_i = D_r G(0, 1)$, $dp_i = D_p G(0, 1)$. Here $G(0, 1)$ are independent Gaussian random numbers with zero mean and standard deviations equal to 1.
- Advancing each time step, a elongated cell move by $dx_i = D_e G(0, 1)$, $dy_e = D_r G(0, 1)$, $dp_i = D_p G(0, 1)$. Here $G(0, 1)$ are independent Gaussian random numbers with zero mean and standard deviations equal to 1.
- If $|p_i| \geq 1$, cell i will switch its phenotype, p_i is reset to 0.

As shown in Fig. S5, the simulation results qualitatively agree with the experiment observations. The agreement supports the key assumption of the model, that cell phenotype

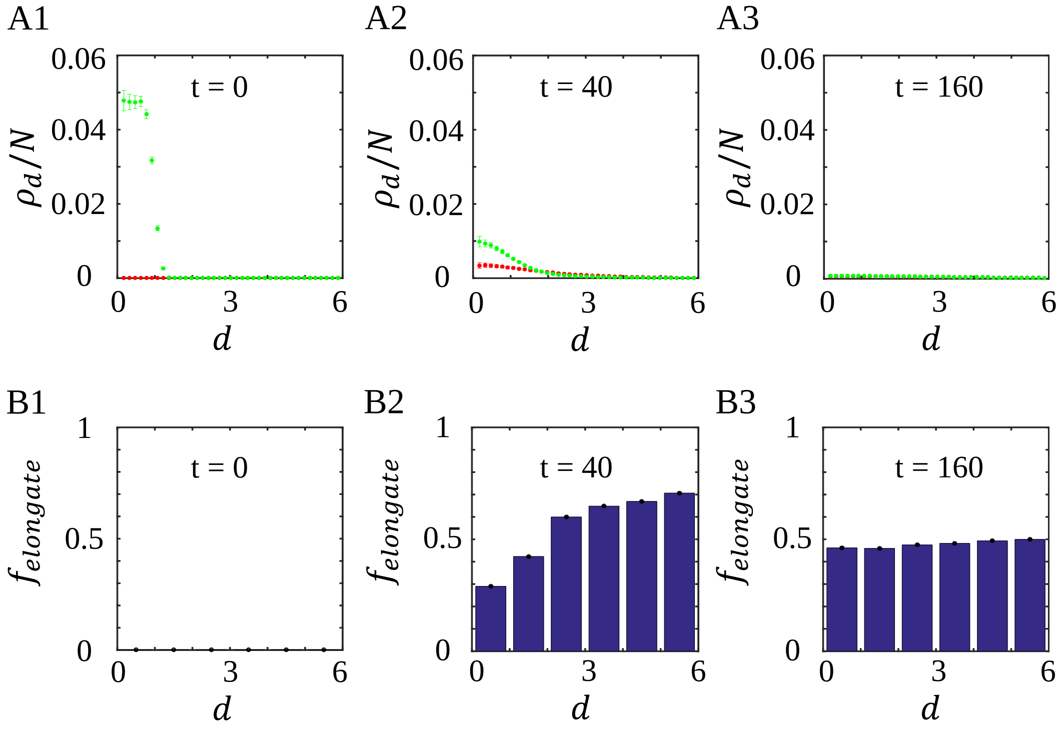

FIG. S5: Stochastic simulation results of collective diskoid invasion. A1-A3: cell density normalized by the total number of cells at varying invasion distance d. Green: rounded; Red: elongated. Errorbars: standard deviation of 100 simulations. B1-B3: fraction of elongated cells at varying invasion distance d. Errorbars: standard error of the mean of 100 simulations. The simulation model parameters are: $D_e = 0.3$, $D_r = 0.1$, $D_p = 0.2$.

switching can be described by stochastic events in a parameter space. We are currently extending the model by introducing cell-cell communications and ECM-induced migration biases. Quantitative analysis of the refined model and its comparison with the experiment results will be reported elsewhere.
